# Evidence for a general stiffening motor control pattern in neck pain: a cross sectional study

**DOI:** 10.1186/s12891-015-0517-2

**Published:** 2015-03-17

**Authors:** Ingebrigt Meisingset, Astrid Woodhouse, Ann- Katrin Stensdotter, Øyvind Stavdahl, Håvard Lorås, Sigmund Gismervik, Hege Andresen, Kristian Austreim, Ottar Vasseljen

**Affiliations:** Department of Public Health and General Practice, Faculty of Medicine, Norwegian University of Science and Technology, Trondheim, Norway; Department of Engineering Cybernetics, Norwegian University of Science and Technology, Trondheim, Norway; Department of Physical Medicine and Rehabilitation, St. Olavs University Hospital, Trondheim, Norway; Department of Neuroscience, Faculty of Medicine, Norwegian University of Science and Technology, Trondheim, Norway

**Keywords:** Neck, motor control, Neck flexibility, Proprioception, Head steadiness, Trajectory movement control, Postural sway, Clinical features

## Abstract

**Background:**

Neck pain is associated with several alterations in neck motion and motor control. Previous studies have investigated single constructs of neck motor control, while few have applied a comprehensive set of tests to investigate cervical motor control. This comparative cross- sectional study aimed to investigate different motor control constructs in neck pain patients and healthy controls.

**Methods:**

A total of 166 subjects participated in the study, 91 healthy controls (HC) and 75 neck pain patients (NP) with long-lasting moderate to severe neck pain. Neck flexibility, proprioception, head steadiness, trajectory movement control, and postural sway were assessed using a 3D motion tracking system (Liberty). The different constructs of neck motion and motor control were based on tests used in previous studies.

**Results:**

Neck flexibility was lower in NP compared to HC, indicated by reduced cervical ROM and conjunct motion. Movement velocity was slower in NP compared to HC. Tests of head steadiness showed a stiffer movement pattern in NP compared to HC, indicated by lower head angular velocity. NP patients departed less from a predictable trajectory movement pattern (figure of eight) compared to healthy controls, but there was no difference for unpredictable movement patterns (the Fly test). No differences were found for postural sway in standing with eyes open and eyes closed. However, NP patients had significantly larger postural sway when standing on a balance pad. Proprioception did not differ between the groups. Largest effect sizes (ES) were found for neck flexibility (ES range: 0.2- 0.8) and head steadiness (ES range: 1.3- 2.0). Neck flexibility was the only construct that showed a significant association with current neck pain, while peak velocity was the only variable that showed a significant association with kinesiophobia.

**Conclusions:**

NP patients showed an overall stiffer and more rigid neck motor control pattern compared to HC, indicated by lower neck flexibility, slower movement velocity, increased head steadiness and more rigid trajectory head motion patterns. Only neck flexibility showed a significant association with clinical features in NP patients.

**Electronic supplementary material:**

The online version of this article (doi:10.1186/s12891-015-0517-2) contains supplementary material, which is available to authorized users.

## Background

Neck pain is common in the general population with one-year prevalence varying from 30% to 50% [1]. Globally, neck pain is the fourth leading cause of years lived with disability, which underlines the importance of research to develop effective prevention and treatment programs based on knowledge of underlying mechanisms of neck pain [2]. A recent paper indicates a close connection between alterations in motor control and pain processing in the brain [3].

Research over the last decade indicates several alterations in neck motor control and sensorimotor entities in subjects with neck pain compared to healthy subjects. Neck pain patients may have delayed onset of deep neck flexors [4], increased activation of superficial neck flexors [5], jerky movement patterns [6], decreased cervical flexor endurance [7], lower movement velocity [8-10], decreased cervical muscle strength [11], reduced trajectory movement control [12], irregular and stiffer movement patterns [13,14], increased postural sway [15,16], and reduced joint position sense[17-19]. However, no single parameter stands out as representing motor dysfunction in the neck and studies typically use a subset of variables that vary between studies [17,20].

Surprisingly, few studies have utilized a comprehensive set of neck movement and motor control tests to contrast patients and healthy subjects. Such comparisons may help identifying specific underlying neck movement or motor control constructs that differentiate patients from healthy subjects. Based on the previous research we decided to group different neck motion and motor control parameters within different constructs of tentative underlying neck motor dysfunction. The aim of this study was thus to compare neck motion and motor control in neck pain patients with moderate to severe neck pain and healthy subjects with tests representing five different constructs: neck flexibility, proprioception, trajectory movement control, head steadiness, and postural sway. Secondary aim was to evaluate the association between clinical features such as pain, disability, and kinesiophobia and the constructs of motor control and neck motion.

## Methods

We conducted a comparative case–control study (n = 166) in the period August 2012 to February 2014. The data from healthy controls (n = 91) were collected by a nurse and a physiotherapist, while the neck pain patients (n = 75) data were collected by a second physiotherapist. The different examiners were equally and well trained in the test procedures. In addition the physiotherapist who performed the data collection for the NP group observed the data collection in the HC group to avoid discrepancies in the procedures. All subjects gave written and informed consent and the study was conducted in accordance with the Helsinki Declaration and approved by the Regional Committee for Medical and Health Research Ethics, REC Central (2011/2522/REC Central).

### Healthy control group (HC)

Men and women between 18–67 years with no neck pain were included in the HC group. The subjects were recruited by inviting friends and colleagues at the local university and university hospital. Exclusion criteria were episode of neck pain within the last 3 months, markedly reduced or uncorrected vision, history of neck trauma, diagnosed with neurological or orthopedic conditions that could affect motor control, positive Spurling’s test for neurological radiating arm pain, pregnancy, or insufficient comprehension of Norwegian.

### Neck pain group (NP)

Neck pain patients were recruited from private physiotherapy clinics in primary health care (56 subjects) and from a specialized neck and back pain clinic at the university hospital (19 subjects). Patients were initially screened for eligibility by telephone. Upon later examination, inclusion criteria were non- traumatic neck pain as the main problem with a score of 3 or more on numerical rating scale (NRS; 0–10) , where 0 represent no pain and 10 worst imaginable pain, at the day of testing and the current neck pain episode lasting >2 weeks. Exclusion criteria were the same as for the control group, except the criteria for neck pain.

### Questionnaire data

On the day of testing, both HC and NP patients first completed a questionnaire which consisted of biographical data (age, gender, height and weight), duration of current neck pain episode, neck pain intensity at the day of testing and average neck pain last month assessed by NRS. Further descriptive data were obtained by the Neck Disability Index (NDI; 0–100) [21], Tampa Scale of Kinesiophobia (TSK; 13–52) [22], Pain Catastrophizing Scale (PCS;0–52) [23], and Pain Self Efficacy Questionnaire (PSEQ;0–60) [24]. High values in TSK and PCS indicate more kinesiophobia and catastrophizing, respectively, while low values in PSEQ indicate low self efficacy.

### Instrumentation and sensors

Motion data for the cervical and postural sway measurements were acquired with body worn sensors using the Liberty electromagnetic motion tracker system (Polhemus, Inc, Colchester, Vermont, USA) with a sampling rate of 240 Hz. Sensor 1 (S1) was placed on the subject’s forehead 1 cm above arcus superciliaris, the second sensor (S2) was placed on the spinous process of Th2, and a third sensor (S3) was placed in the area of the spinous processes of L4-L5. Tight elastic bands were used to hold the sensors in position. The electromagnetic transmitter (TX) was positioned at a distance of 10–50 cm above the head during all the measurements. For S1 and S2 raw data were low pass filtered at 20 Hz using a 2nd order Butterworth filter, while raw data for S3 were low pass filtered at 5 Hz.

A software tool based on Matlab (The MathWorks, Inc., Natick, MA, USA) was developed (SINTEF ICT, Applied Cybernetics and Dept. of Engineering Cybernetics, NTNU, Norway) to record and analyze the motion data. Table 1 shows the sensors used for calculations of the different tests. The coordinate system defined by the TX was used for calculating all variables except cervical range of motion (ROM). For this variable, a new coordinate system was calibrated for each subject to adjust the coordinate axes to the individually preferred axes of cervical motion. Detailed description of the calibration is available in Additional file 1.

**Table 1:** **Description of the motor control and cervical motion variables**

**Construct**	**Test**	**Assessment**	**Unit of measure**	**Sensors**	**Reps per test**	**Analyzed**	**Comments**
Neck flexibility	Active neck movements in flexion/extension, rotation and lateral flexion	Cervical ROM in flexion/extension, rotation, and, lateral flexion	deg	S1 vs S2	3	Avg	Full cycle cervical ROM
		Conjunct motion in the two associated movement planes	deg			Avg	According to Woodhouse et al. (2008)
		Peak velocity in flexion/extension, rotation and lateral flexion	deg/s			Avg	3 D angular velocity
Proprioception	Joint position error in left and right head rotation	3D repositioning error in left and right rotation	deg	S1 vs S2	6	Avg	3 repetitions in each direction
Trajectory movement control	FOE slow speed	Average point deviation (PD)	cm	S1 vs TX	1	Single	30 sec duration
	FOE fast speed				1		20 sec duration
	FOE in standing, slow speed				1		30 sec duration.
	The Fly test, 1A	Average point deviation (PD)	cm	S1 vs TX	1	Single	30 sec duration for all of the Fly tests. Adopted from Kristjansson et al. (2010)
	The Fly test, 2B				1		
	The Fly test, 1B				1		
	The Fly test, 2A				1		
Head steadiness	Isometric neck flexion, low load	Average 3 D angular velocity	deg/s	S1 vs S2	1	Single	60 sec duration 60° recumbent position
	Isometric neck flexion, high load	Average 3 D angular velocity	deg/s		1		30 sec duration Supine position
		Holding time	sec				
Postural sway	Standing balance EO	Sway area (95 % confidence area)	cm^2^	S3 vs TX	1	Single	60 sec duration for the tests of standing balance.
	Standing balance EC				1		
	Standing balance EOB				1		
	FOE in standing				1		30 sec duration

The column test lists the order of the tests in the data collection.

ROM = range of motion. CM = conjunct motion. Deg = degrees. 3D = 3 dimensional. FOE = figure of eight. EO = eyes open. EC = eyes closed. EOB = eyes open balance pad. S1 = forehead sensor. S2 = spinous process of T2. S3 = spinous process of L4-L5. TX = transmitter on Liberty. 1A = easy pattern, small ROM. 1B = easy pattern, large ROM. 2A = difficult pattern, small ROM. 2B = difficult pattern, large ROM.

### Outcome variables and testing procedures

The description of the tests of motor control and the calculated variables are summarized in Table 1. We adopted five constructs of motor control to group the different tests used in the study. Standardized instructions were used for all tests. The tests were performed in the order listed in Table 2.

**Table 2:** **Subject characteristics and clinical features**

	**Neck pain patients n = 75**	**Healthy controls n = 91**	**p**
Age	43.1 (12.9)	40.8 /13.8)	0.28
Gender (male/female	20/55	43/48	0.01
Body mass index	24.9 (4.7)	25.0 (3.5)	0.9
Current neck pain (NRS: 0-10)	4.6 (1.4)	N/A	
Worst neck pain last month (NRS: 0-10)	7.4 (1.5)	N/A	
Duration of neck pain, n (%)		N/A	
< 3 months	7 (9%)		
3-6 months	13 (17%)		
> 6 months	55 (74%)		
Neck Disability Index (0-100)	31.2 (11.6)	N/A	
TSK (13-52)	24.4 (4.3)	N/A	
PCS (0-52)	12.9 (8.5)	N/A	
PSEQ (0-60)	44.3 (10.0)	N/A	
Concurrent low back pain, n (%)	20 (27%)	N/A	
Self-rated general health, n (%)		N/A	
Poor	0 (0)		
Fair	36 (48%)		
Good	37 (49%)		
Very good	2 (3%)		
Frequency physical activity, n(%)		N/A	
< once per week	10 (12%)		
1-3 days per week	51 (68%)		
4-7 days per week	14 (19)		
Use of analgesica, n (%)	38 (51)	N/A	

Mean (SD), unless otherwise stated.

NRS = Numerical Rating Scale. TSK = Tampa Scale of Kinesiophobia. PCS = Pain Catastrophizing Scale. PSES = Pain Self Efficacy Questionnaire. N/A = Not applicable.

### Neck flexibility

Maximal cervical ROM was measured with the subjects seated on a wooden bench with backrest in 80^0^ recumbent position and the shoulders fixed with nonflexible shoulder straps to avoid movement of the thorax. The subjects were asked to move as far as possible in all three primary movement planes (flexion/extension, rotation in the horizontal plane and lateral flexion) with a self- preferred velocity. Start and stop of the recording was manually set by the examiner before and after the movement. Maximal cervical ROM was calculated as the mean of three trials for each primary movement plane. During each primary plane ROM test neck flexibility was also assessed by the degree of motion in accessory planes, i.e., conjunct motion (CM), which was calculated as the maximum ROM in the two associated movement planes, adopted from Woodhouse et al. [14]. Peak velocity in the three tests of maximal cervical ROM was computed to assess movement velocity (see Additional file 1), since this variable has been shown to differentiate neck pain patients from healthy controls [8,10].

### Proprioception

Joint position error (JPE) was used to assess proprioception and was recorded as the difference in head orientation at neutral position before and after cervical left and right head rotation (see Additional file 1). The subjects were blind- folded and instructed to start with their head in a preferred neutral position and then rotate the head as far as possible and back to the neutral position. The subjects performed three repetitions in each direction; first to the left and then to the right. Subjects were asked to respond orally when they believed they had returned to neutral position. The examiner did not reposition the subjects’ head back to the initial neutral position, but used the end position of the previous trial as the starting position for the next repetition.

### Trajectory movement control

**Figure 1:**
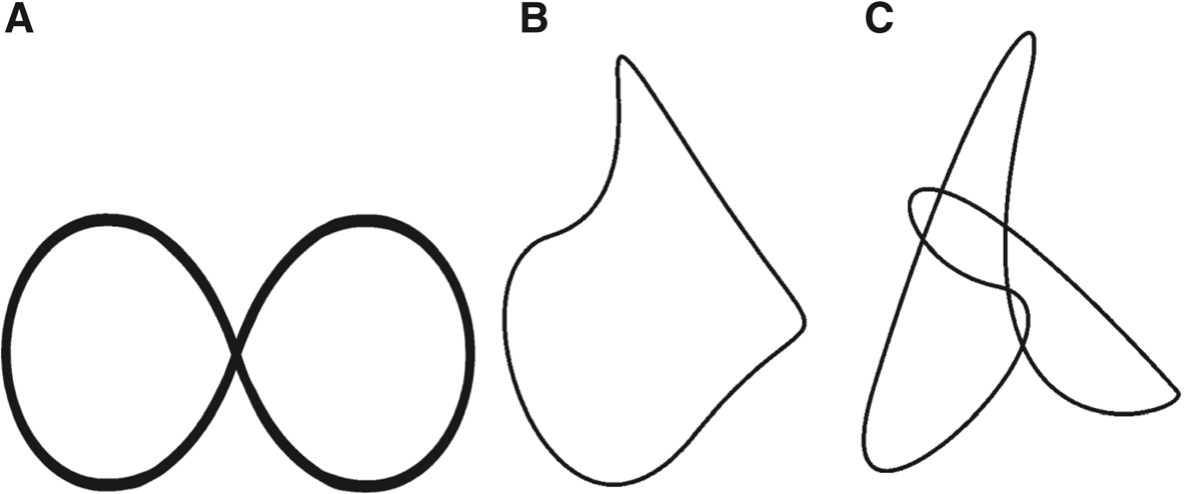
**The movement patterns of the figure of eight and “the Fly” tests for assessing trajectory movement control. A**: Figure of eight. **B**: Movement pattern 1A and 1B in the Fly test C: Movement pattern 2A and 2B in the Fly test (adopted from Kristjansson et al. (2010)). Mean cervical ROM in the performance of the figure of eight was 11° flexion/extension and 24° rotation in the horizontal plane. Mean cervical ROM in the Fly test was 14° flexion/extension and 10° rotation in the horizontal plane in movement pattern 1A, and 15° flexion/extension and 10° rotation in the horizontal plane in movement pattern 2A. Movement pattern 1B and 2B, which was the same patterns as 1A and 2A, respectively, had twice as much cervical ROM (movement ratio head to cursor 2:1).

Two head tracking tasks were performed in order to assess trajectory movement control; a figure-of-eight test (FOE) (adopted from the study of Woodhouse et al. [25]) and a “Fly test” (as described by Kristjannson et al. [12]). For the FOE test, a horizontal figure-of- eight was displayed on a screen in front of the subjects at a distance of 250 cm (Figure 1). The subjects were instructed to follow a white cursor moving along the line of the figure as accurately as possible by moving their head. The speed of the tracking was set by the movement of the white cursor. The movement of the head was projected on the screen as a red cursor. Two different tracking velocities, slow and fast, were used to investigate possible differences in the speed-accuracy trade-off between NP subjects and HC’s [26]. For the slow and fast tracking velocity tests the speed of the white cursor displayed on the screen was approximately 10 cm/s and 15 cm/s, respectively. The head velocity required to track the FOE was approximately 3.0 °/s and 4.3°/s for the slow and fast tracking velocity, respectively. The test with low velocity was also repeated in standing. The HC and NP subjects were familiarized with the test by performing one test session with high velocity.

Two patterns of the Fly test (Figure 1) were used in this study corresponding to the easy (1A and 1B) and medium (2A and 2B) patterns described by Kristjanssons et al. [12]. Two different demands for head movement were applied during the Fly test, one with a head to projected cursor movement ratio of 1:1 (1A and 2A) and another with a ratio 2:1 (1B and 2B), the latter implying that the head had to move twice as far to move the cursor on the screen compared to the first test. The 2:1 ratio was included to study demands of increased ROM on trajectory movement control. The tracking velocities for the Fly tests measured in head motion velocity were in the range of 2–5 °/s. The patterns were not visible to the subject and thus unpredictable. The setup was similar to the figure of eight test and subjects were instructed to follow the white cursor (“the Fly”) as accurately as possible. The HC were familiarized with the test by performing one of the fly tests, and the NP performed two of the tests. Point deviation (PD), a measure of movement accuracy, was calculated as the mean absolute distance (cm) between the red cursor and the white cursor both during the FOE test and the Fly test.

### Head steadiness

Isometric neck flexion (INF) was used to investigate the ability to hold the head steady under two conditions, low load and high load. For the low load test the subjects were seated in a 60 ° recumbent position with footrest and without shoulder straps. They were asked to slightly lift their head (1-2 cm) from the backrest and hold their head as steady as possible in the same position for 1 minute. For the INF high load test the subjects were positioned in supine and were asked to do craniocervical flexion while their head was positioned on the bench, then to lift their head slightly from the bench and hold their head as steady as possible in the same position for 30 s. The test was ended if the subjects touched the table with the back of their head or if the subjects chose to end the test due to fatigue or neck pain. Angular velocity of the head was calculated to assess the ability to hold the head steady during the INF test. Holding time during the high load INF test was used as a descriptive variable. Angular velocity (deg/s) was calculated as the point to point change in orientation of the forehead sensor (S1) over time, relative to the sensor placed on the spinous process of T2 (S2). Holding time in the INF tests was registered with a stopwatch.

### Postural sway

Postural sway in quiet standing was assessed during 60 s for each one of four conditions. The first condition was a dual task where the FOE test with low tracking velocity was performed during quiet standing. In the second condition eyes open (EO) the subjects were instructed to focus at a point on the wall 250 cm straight in front of them. For the third condition subjects were blindfolded (EC). The fourth and last condition was performed with eyes open standing on a balance pad (EOB). Unfortunately, postural sway during the FOE in standing was not recorded in 43 subjects in HC, leaving only 48 subjects in the HC group for this analysis. The same standing position was ensured for all conditions (feet parallel with 10 cm between the medial malleoli and arms held across the chest) and the order of presentation of conditions was the same for all subjects. The instructions given were to “stand still for one minute”. Sway was assessed from the antero-posterior and the mediolateral position data from the sensor (S3) placed on spinous process of L4-L5 and 95% confidence interval for sway area (cm^2^) was calculated.

### Statistical analysis

Outliers, those who did not perform the test correctly or were exposed to technical problems were dropped from the analyses. The number of subjects analyzed for the different variables are reported in Tables 3 and 4.

**Table 3:** **Group comparisons of neck flexibility and proprioception**

	**Neck pain (n = 75)**	**Healthy controls (n = 91)**	**p**
**Neck flexibility**			
**Flexion/extension (°)** ^1^	110.1 (105.7-114.5)	126.2 (122.3-130.2)	<0.01
CM (°)^1^	12.3 (11.3-13.3)	16.5 (15.6-17.4)	<0.01
CM (°)^2^	12.9 (11.9-14.0)	16.0 (15.1-16.9)	<0.01
CM (°)^3^	13.7 (12.4-15.0)	15.4 (14.4-16.3)	0.03
Peak velocity (°/s)^1^	70.6 (62.5-78.7)	115.6 (108.4-122.8)	<0.01
Peak velocity (°/s)^2^	75.0 (66.8- 83.1)	112.1 (104.9-119.4)	<0.01
**Rotation (°)** ^1^	128.2 (124.3-132.2)	140.7 (137.2-144.2)	<0.01
CM (°)^1^	19.8 (18.0-21.6)	25.1 (23.5-26.7)	<0.01
CM (°)^2^	20.6 (18.7-22.4)	24.5 (22.8-26.1)	<0.01
CM (°)^3^	21.1 (19.2-23.1))	24.0 (22.3-25.7)	0.04
Peak velocity (°/s)^1^	109.3 (98.9-119.7)	158.9 (149.6-168.3)	<0.01
Peak velocity (°/s)^2^	114.3 (103.9-124.7)	154.9 (145.7-164.2)	<0.01
**Lateral flexion (°)** ^1^	68.1 (64.7-71.6)	72.6 (69.5-75.7)	0.06
CM (°)^1^	45.7 (40.1-51.3)	62.5 (57.5-67.5)	<0.01
CM (°)^2^	44.9 (39.4- 50.5)	63.1 (58.1-68.0)	<0.01
CM (°)^3^	52.7 (47.6-57.7)	56.9 (52.4-61.4)	0.25
Peak velocity (°/s)^1^	57.9 (52.2-63.5)	85.7 (80.6-90.7)	<0.01
Peak velocity (°/s)^2^	58.6 (53.0- 64.2)	85.1 (80.1-90.1)	<0.01
**Total cervical ROM (°)** ^1^	306.5 (296.5-316.5)	339.5 (330.6-348.5)	<0.01
**Proprioception**			
JPE (°)^2^	5.6 (5.2-6.1)	5.1 (4.6-5.5)	0.11

Maximal cervical ROM, conjunct motion and peak velocity in the three primary neck movement planes.

Given values are mean (95 % CI) adjusted for 3 different models of covariates.

^1^ Adjusted for age and gender (model 1).

^2^ Adjusted for age, gender, and cervical ROM (model 2).

^3^ Adjusted for age, gender, cervical ROM, and peak velocity (model 3).

p = p- value. ROM = range of motion. CM = conjunct motion in the two accessory movement planes. JPE = joint position error.

**Table 4:** **Group comparisons of head steadiness, trajectory movement control, and postural sway**

	**Neck pain**	**Healthy controls**	**n=**NP/HC	**p** ^**1**^
**Head steadiness**				
**INF Low load**				
Angular velocity (°/s)	1.3 (1.2-1.4)	1.7 (1.6-1.8)	75/90	<0.01
**INF High Load**				
Angular velocity (°/s)	2.8 (2.6-2.9)	4.5 (4.3-4.7)	73/91	<0.01
**Trajectory movement control**				
**FOE Low speed**				
Point deviation (cm)	3.4 (3.1-3.7)	3.8 (3.4-4.1)	75/89	0.17
**FOE High speed**				
Point deviation (cm)	4.4 (4.1-4.8)	5.2 (4.8-5.6)	75/91	<0.01
**FOE Standing, low speed**				
Point deviation (cm)	2.9 (2.6-3.1)	3.3 (3.0-3.6)	74/91	0.02
**The Fly test**				
PD test 1A (cm)	2.2 (2.0-2.4)	2.5 (2.3-2.7)	75/89	0.03
PD test 1B (cm)	2.1 (2.0-2.2)	2.1 (2.0-2.3)	75/89	0.63
PD test 2A (cm)	3.1 (2.9-3.3)	3.3 (3.1-3.5)	74/88	0.33
PD test 2B (cm)	2.8 (2.7-3.0)	2.8 (2.6-2.9)	75/83	0.64
**Postural sway**				
Sway area EO (cm^2^)	3.0 (2.4-3.5)	2.7 (2.2-3.3)	72/90	0.53
Sway area EC (cm^2^)	2.5 (2.0-3.1)	2.0 (1.7-2.3)	72/87	0.12
Sway area EOB (cm^2^)	11.0 (9.7-12.3)	8.1 (7.4-8.7)	73/91	<0.01
Sway area FOE (cm^2^)	4.3 (3.3-5.2)	5.9 (4.2-7.5)	73/48	0.09

Given values are mean (95 % CI) adjusted for age and gender (model 1).

^1^ p value adjusted for age and gender (model 1).

INF = isometric neck flexion. FOE = figure of eight. PA = point deviation. 1A = easy pattern, small ROM.

1B = easy pattern, large ROM. 2A = difficult pattern, small ROM. 2B = difficult pattern, large ROM.

EO = eyes open. EC = eyes closed. EOB = eyes open balance pad.

We used the chi square test to analyze baseline group differences for categorical variables. Multiple regression was used to investigate group differences for cervical ROM, CM, peak velocity, and JPE. Multiple robust regression with Huber’s method was used for the other variables due to heteroskedasticity [27]. We adjusted for age and gender (model 1) in all analyses because age has been shown to influence several of the variables measured [28-30] and gender was not equally distributed between the groups (Table 1). In the analyses of CM we also adjusted for maximum ROM in the primary plane (model 2) and in the final model for both maximum ROM and peak velocity in the primary plane ROM test (model 3), since these covariates are shown to influence smoothness of movement in previous studies [31]. In the sole analysis of peak velocity and JPE we adjusted for maximum ROM [6]. All the variables, except ROM and CM, showed skewness of the data. Log- transformation of these variables gave acceptable normal distribution, but did not change the result of the regression analysis, thus, non-transformed data and p- values are reported for ease of interpretation. To evaluate the association between clinical parameters (NRS, NDI, duration of current neck pain episode, and TSK) and the constructs of motor control in the NP group we used correlation analysis. For the continuous variables NPRS, NDI, and TSK we used Pearson’s r and for the categorical variable duration we used Spearman’s rho. Since tests within each construct were highly correlated and to avoid too many results, we chose the tests with largest effect sizes in the correlation analysis. Effect size (ES) was calculated with this formula: $ES=\frac{H{C}_{mean}-N{P}_{mean}}{\sqrt{\frac{H{C}_{S{D}^2}+N{P}_{S{D}^2}}{2}}},$ where SD is the standard deviation [32]. Calculation of 95 % CI for the ES was done by first calculating the variance $\left({S}_{ES}^2\right)$ for the sampling distribution of the effect size:

${S}_{ES}^2=\frac{H{C}_n+N{P}_n}{H{C}_n\times N{P}_n}+\frac{E{S}^2}{2\Big(H{C}_n+N{P}_n},$ where n is the group size. Then the 95 % CI was calculated using the formula according to Fritz et al. [32]: 95 % *CI* = *ES* ± *z*_0.025_*s*_*ES*_The significance level was defined as p < 0.05. All statistical analyzes were performed using STATA 13 (Stata Corp., College Station, TX, USA).

## Results

The groups were similar in age and BMI, but there were a higher proportion of women in the HC group (Table 2). The NP group had a mean neck pain score of 4.6 on NRS at the day of testing and 7.4 on NRS for worst neck pain last month. A large proportion of the NP patients (74%) stated that their neck pain started more than 6 months ago (Table 2). The NP subjects showed a moderate disability measured by the NDI (mean 31.2; SD 11.6), a moderate kinesiophobia measured by the TSK (mean 24.4; SD 4.3), and a moderate pain catastrophizing measured by the PCS (mean 12.9; SD 8.5). Other characteristics of the NP group are shown in Table 2. Nine NP subjects were excluded due to NRS <3 for neck pain on the day of testing.

### Neck flexibility

**Figure 2:**
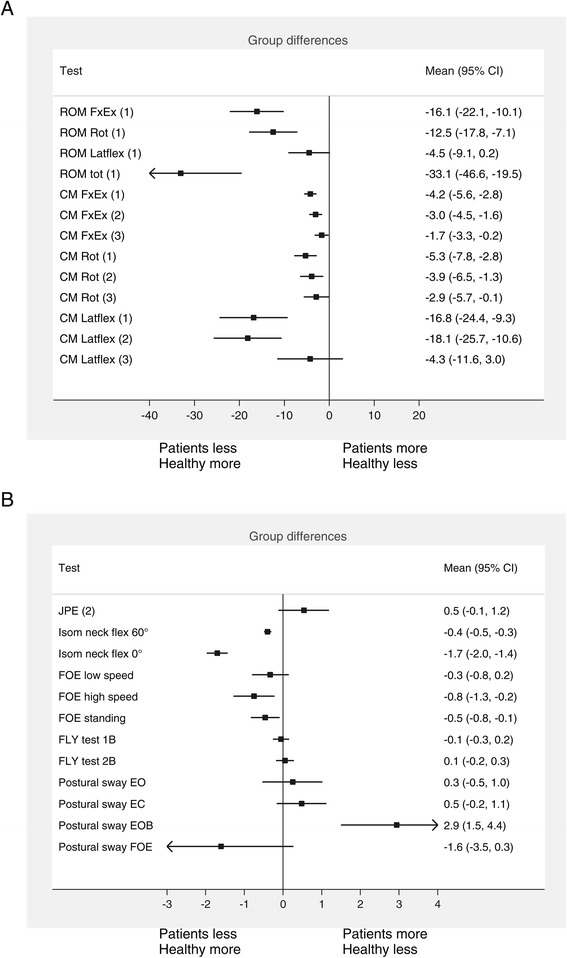
**Forest plot of the group difference (95 % CI) between neck pain patients and healthy controls.** Number in parentheses behind test variables states the analytic model applied. Analysis were adjusted for age and gender (model 1), plus cervical ROM (model 2), plus peak velocity (model 3). All variables in B are adjusted for model 1, except for JPE which is adjusted for model 2. ROM = range of motion. FxEx = flexion/extension. Latflex = lateral flexion. JPE = joint position error. FOE = figure of eight. Fly test 1B = easy pattern, large ROM. Fly test 2B = difficult pattern, large ROM. EO = eyes open. EC = eyes closed. EOB = eyes open balance pad.

NP patients had significantly less maximal cervical ROM in flexion/extension and rotation compared to HC after adjusting for age and gender, while lateral flexion barely fell short of reaching significance (Table 3 and Figure 2A). Summing ROM in the three primary planes in total ROM showed a difference of 33.1° (95% CI; −46.6,-19.5; p < 0.001) between the two groups (Figure 2A). There was no significant gender difference in total cervical ROM or when the primary planes were analyzed separately. Peak velocity during all ROM tests was significantly lower in NP compared to HC, and remained significantly lower also after adjusted for cervical ROM in the primary plane (Table 3). CM in accessory planes during all primary planes motion was significantly smaller in the NP patients compared to HC (Table 3). The differences remained significant after adjusting for maximum ROM in the primary plane. When adjusted for peak velocity CM in flexion/extension and rotation in the NP groups were still significantly smaller compared to HC, but not for CM in lateral flexion (Table 3).

### Proprioception

There was no significant between group difference (p = 0.11) in relocation error in the JPE test (Table 3).

### Head steadiness

In the low load and high load tests NP patients had markedly lower head angular velocity compared to HC. In the low load test the mean group difference was −0.4 °/s (95% CI; −0.5 to −0.3; p < 0.001) and in the high load test −1.7 °/s (95% CI; −2.0 to −1.4; p < 0.001). Largest effect sizes were found for head steadiness and neck flexibility (Table 5). All subjects in the HC group were able to hold for 60 s in the low load and 30 s in the high load test, whereas 8 subjects in NP group did not manage to hold their head for 30 s in the high load test. Two of these patients had holding time <3 s in the high load test and were therefore excluded from the calculations of the kinematic variables.

**Table 5:** **Summary of main results**

	**Indicate less motion**	**Normal**	**Indicate larger motion**	**Effect size Mean (95 % CI)**
**Neck flexibility**				
**Flexion/extension** ^1^	X			0.84 (0.52 to 1.16)*
CM^3^	X			0.37 (0.06 to 0.68)*
Peak velocity^2^	X			1.05 (0.72 to 1.37)*
**Rotation** ^1^	X			0.72 (0.41 to 1.04)*
CM^3^	X			0.36 (0.05 to 0.67)*
Peak velocity^2^	X			0.90 (0.57 to 1.22)*
**Lateral flexion** ^1^		X		0.30 (-0.01 to 0.61)
CM^3^		X		0.19 (-0.11 to 0.50)
Peak velocity^2^	X			1.08 (0.75 to 1.41)*
**Total cervical ROM** ^1^	X			0.76 (0.44 to 1.08)*
**Proprioception**				
JPE^2^		X		-0.26 (-0.56 to 0.05)
**Head steadiness**				
Low load^1^	X			1.29 (0.95 to 1.63)*
High Load^1^	X			1.95 (1.58 to 2.32)*
**Trajectory movement control**				
FOE low speed^1^		X		0.22 (-0.09 to 0.53)
FOE high sped^1^	X			0.45 (0.13 to 0.76)*
FOE standing^1^	X			0.40 (0.09 to 0.71)*
Fly test 1A^1^	X			0.36 (0.05 to 0.68)*
Fly test 1B^1^		X		0.07 (-0.23 to 0.38
Fly test 2A^1^		X		0.16 (-0.15 to 0.47)
Fly test 2B^1^		X		-0.07 (-0.39 to 0.24)
**Postural sway**				
Open eyes^1^		X		-0.09 (-0.40 to 0.21)
Closed eyes^1^		X		-0.26 (-0.57 to 0.06)
Balance pad^1^			X	-0.64 (-0.95 to -0.32)*
FOE standing^1^		X		0.30 (-0.07 to 0.66)

Effect sizes for each variable with 95% CI are listed below.

Interpretation of the effect size: 0.2 = small, 0.5 = medium, >0.8 = large.

Negative or positive effect sizes indicate that the NP group has larger or smaller values, respectively, compared to HC. Statistically significant and effect sizes >0.5 are marked with * and bold numbers, respectively.

^1^ Adjusted for age and gender (model 1).

^2^ Adjusted for age, gender, and cervical ROM (model 2).

^3^ Adjusted for age, gender, cervical ROM, and peak velocity (model 3).

* = p value <0.05.

ROM = range of motion. CM = conjunct motion in the two accessory movement planes. JPE = joint position error. INF = isometric neck flexion. FOE = figure of eight. 1A = easy pattern, small ROM. 1B = easy pattern, large ROM. 2A = difficult pattern, small ROM. 2B = difficult pattern, large ROM. EO = eyes open. EC = eyes closed. EOB = eyes open balance pad.

### Trajectory movement control

Table 4 shows that HC subjects departed more from the trajectory pattern in the FOE test than the NP subjects, indicated by the higher point deviation values. The differences were statistical significant for the high speed FOE test with a mean group difference in PD of −0.8 cm (95% CI;-1.3 to −0.2; p < 0.01) and the FOE test in standing (mean difference: −0.5 cm; 95% CI; −0.8 to −0.1; p < 0.05), (Figure 2B). HC also showed more trajectory departure (i.e. higher point deviation) in the Fly test 1A compared to the NP group (mean difference: −0.3 cm; 95 % CI; −0.6 to −0.03; p < 0.05). None of the other movement patterns in the Fly tests revealed any significant group differences in PD between the NP group and HC (Table 4).

### Postural sway

Postural sway during quiet standing with EO and EC did not differ significantly between the groups (Figure 2B and Table 4). The NP group had a significant larger sway area for the EOB test compared to HC (mean difference: 2.9; 95% CI; 1.5 to 4.4; p > 0.01). Contrary, the NP patients had less sway area during the FOE test where subjects had to perform neck motion during the standing balance test, but this difference was not statistically significant (mean difference: −1.6; 95 % CI; −3.5 to 0.3; p = 0.09).

### Associations between clinical features and constructs of motor control

Neck flexibility was the only construct that was significantly associated with clinical features, but the associations were weak. Current neck pain was significantly associated with ROM in flexion/extension (r = −0.36; p < 0.01), CM (r = −0.26; p < 0.05), and peak velocity (r = −0.34; p < 0.01) during flexion/extension (Table 5). TSK was significantly correlated with peak velocity in flexion /extension (r = 0.23; p < 0.05). NDI and duration of current neck pain episode were not significantly associated with neck flexibility (Table 6). The Fly test showed a significant correlation with NDI (r = 0.27; p < 0.05), but not for the other clinical features.

**Table 6:** **Correlations between clinical parameters and constructs of motor control and neck motion in neck pain patients (n = 75)**

Variables	**NPRS r**	**NDI r**	**Duration** ^**1**^ **rho**	**TSK r**
**Neck flexibility**				
Flexion/extension	-0.36**	-0.10	-0.09	-0.11
Conjunct motion	-0.26*	0.05	-0.09	0.12
Peak velocity	-0.34**	-0.21	-0.14	0.231*
**Proprioception**				
Joint position error	0.01	0.147	0.03	0.12
**Head steadiness**				
INF low load	-0.1	-0.13	-0.11	-0.11
INF high load	0.05	-0.12	-0.05	-0.06
**Trajectory movement control**				
PD FOE fast	0.18	0.09	-0.14	-0.02
PD Fly test^2^	0.19	0.27*	-0.21	-0.10
**Postural sway**				
Area EOB	0.03	-0.04	0.14	-0.07

Clinical parameters are current neck pain measured by NRS, neck disability index (NDI), duration of current neck pain episode, and kinesiophobia measured by TSK. Given values are Pearson’s r for NRS, NDI and TSK and Spearman’s rho for duration with corresponding p-values. Correlation coefficients with p < 0.05 are in bold.

^1^ Duration of pain, five categories from short to long duration of current neck pain.

^2^ Fly test with easy pattern and small neck range of motion.

* = p value < 0.05. ** = p value < 0.01.

NRS = numerical rating scale. NDI = Neck Disability Index. TSK = Tampa Scale of Kinesiophobia. INF = isometric neck flexion. PD = point deviation. FOE = figure-of-eight. EOB = eyes open balance pad.

## Discussion

Overall, this study points to an altered neck motor control in patients with moderate to severe neck pain with long duration. NP patients had less cervical ROM, reduced conjunct motion, lower peak velocity during cervical ROM tests, less head motion in the isometric head flexion steadiness tests, showed more “rigid” trajectory head motion patterns and more postural sway in standing on a balance pad. Except for the latter, all tests may express a general finding of stiffer and more rigid neck motor patterns in neck pain patients (Table 5).

### Neck flexibility

In agreement with other studies [14,33], we found that NP subjects had clearly less neck flexibility in primary cervical planes compared to HC. Also, the finding of lower peak velocity in tests of cervical ROM among NP subjects compared to HC is in accordance with other studies of movement velocity [8,10]. CM can be perceived as a measure of freedom or smoothness of motion during tests of maximal ROM in the cervical cardinal planes. CM has previously been shown to differentiate neck pain patients from healthy controls [10,14], in agreement with this study showing significantly less CM or stiffer movement during all primary planes. A strength of this study is that we controlled for cervical ROM and movement velocity. A recent study showed that smoothness of movement was strongly related to velocity in unconstrained head movements [31]. Less smooth movements may therefore be a result of lower velocity and not altered motor control, which implies that statistically adjusting for movement velocity is imperative when investigating smoothness of movement in unconstrained neck movements [31]. In line with this, group differences in CM in lateral flexion were no longer significant after adjusting for movement velocity. However, reduced CM in flexion/extension and rotation remained significant after adjusting for velocity, indicating that reduced CM is a robust sign of stiffer movement patterns in neck pain patients for these movement planes.

The etiology of the lower peak velocity in NP subjects compared to HC in performing unconstrained neck movements is largely unknown. Vikne et al. suggested that altered muscle activation patterns may not explain the lower peak velocity, since they found that EMG amplitude in neck muscles during unconstrained neck movements was similar between neck pain subjects and healthy controls when adjusted for ROM and movement velocity [9]. On the other hand, they found a negative correlation between fear avoidance and peak velocity measured in the sagittal plane (r value range −0.67 to −0.77), suggesting that cognitive factors might act as critical effect modifiers in the relationship between neck motor control and neck pain. Interestingly, our study confirmed that peak velocity in flexion/extension was significantly associated with kinesiophobia . However, we did not find the same association for peak velocity in rotation and lateral flexion in a secondary analysis, suggesting that movement in flexion/extension might be more related to kinesiophobia in neck pain patients. Other studies that have reported on the association between kinesiophobia and neck flexibility have included whiplash patients [34] or a combination of non-trauma and whiplash patients [35] making comparisons across studies difficult.

### Head steadiness

Different tests of INF have been used in previous studies to investigate holding time [7,36,37], muscle activation patterns [38,39], head steadiness [36], and cervical muscle strength [40]. The present study used head angular velocity to investigate head steadiness, previously described in Woodhouse et al. [36]. Our result of less angular velocity in NP patients compared to HC supports the findings of Woodhouse et al., where the NP group showed a trend of less angular velocity compared to healthy controls. While Woodhouse et al. used only one sensor on the forehead (i.e. S1), we used two sensors to be able to separate head motion from upper body motion, and subjects in our study were also instructed to do craniocervical flexion in the INF test. Lower head angular velocity may indicate that NP patients stiffen their neck to avoid painful movement of the head, possibly due to increased muscle activation in superficial neck muscles to compensate for reduced activation of deep neck muscles [41], or increased co-contraction of cervical agonist–antagonist muscles to increase stability of the cervical spine [42]. Muscle activation was however not recorded in the present study.

### Trajectory movement control

The FOE and the Fly test can be seen as tests of trajectory movement control, because both require continuous feedback from neck mechanoreceptors, visual and vestibular systems [43]. The Fly test has shown good reliability and discriminant validity [12]. Except for one, none of the Fly tests in this study differed significantly between the NP subjects and the HC group. Increasing the ROM demands during the Fly test did not change the results. Previous studies of trajectory movement control using the Fly test found a consistently larger deviation in trajectory movement control in neck pain subjects compared to healthy [12,43]. This was not supported by our findings. The NP subjects in the present study, compared to the non-trauma group in Kristjansson et al. [12], were similar in age and gender distribution, but had a higher score on NDI, indicating more disability. Kristjansson et al. had more men than women in the healthy control group compared to the non-trauma group and whiplash group which had more women. In the HC group we found that females had a consistently larger deviation in the Fly tests compared to men in the HC group suggesting that the group differences in Kristjansson et al. [12] may be influenced by the different gender distribution in the groups. However, Oddsdottir et al. found similar results between healthy females and men for the same Fly tests [29]. This discrepancy between the studies requires further investigation before this test can be implemented in clinical practice. The FOE test, which required a larger displacement in the horizontal plane (Figure 2), showed that NP subjects tended to depart less from the trajectory path compared to HC, a finding that was clearly significant during the fast speed FOE and the FOE in standing tests. Overall, the results from these tests of trajectory movement control indicated an altered motor control strategy by stiffening of dynamic head motion (Table 6).

### Postural sway

We found no difference in postural sway in standing, when the head was kept in a static position, with eyes open and eyes closed. In a review, the majority of studies of non- traumatic neck pain patients did not find altered postural sway across different standing positions and visual conditions [44]. However, NP patients had a markedly larger sway area in the more challenging standing on a balance pad, but a trend of less postural sway when simultaneously performing a dynamic head motion test (FOE) when standing on firm surface. This indicates that stiffening motor control patterns appear only in tests that challenge the neck directly. However, this inference is based on a single postural sway test, and needs further investigation. We speculate that overall stiffening strategy of the body during the FOE test might stem from distraction caused by the dual task. A study of subjects with low back pain showed that a cognitive dual-task reduced postural sway while healthy controls increased postural sway [45]. However, Dualt et al. found that healthy also have less postural sway when performing dual task compared to single tasks [46].

### Proprioception

Tests of JPE are used in studies to evaluate proprioception. Several studies are in agreement with no deficit in proprioception measured by JPE in NP compared to healthy subjects [14,47,48]. However, other have found that neck pain subjects had significantly larger repositioning error [49], or significantly larger variable error compared to healthy subjects [6]. Discrepancies between the studies in the calculation of JPE and test procedures used may partly explain the conflicting evidence. We therefore reanalyzed the data using repositioning error in the primary plane of motion [49] and the variable error, which is a measure of the variability of the repositioning [6]. The analysis revealed no significant difference between the groups, in agreement with our results for the absolute error. However, other tests of proprioception might be more relevant in NP subjects, since a main criticism of the test used in the present study is that the vestibular system may mask important deficits in afferent input from mechanoreceptors in the neck [18].

### Theory of motor control

Hodges & Tucker presented a new theory of motor control adaptation to pain which concurs with the general finding of stiffer and more rigid neck motor control patterns (Table 5) in the present study [50]. The theory emphasizes increased stiffness as an important motor adaptation to acute pain to protect or avoid movement of a painful body part. However, after the acute stage protective stiffening may no longer serve a purpose. The long term consequences of increased stiffness may be decreased movement and movement variability, and consequently increased load on the spine [50]. We do not know if stiffening motor control patterns is a local or a general feature, but our results indicate that stiffening is confined to the local painful area and not a general feature, as NP patients had less stiffening (i.e. more sway) in standing balance compared to HC subjects (Table 5). Tests of neck proprioception were non- significant in this study, indicating that central rather than peripheral mechanisms are involved. However, we do not know if this reflects centrally driven neurophysiological activations or a cognitive response (fear avoidance, catastrophizing etc.), since our study showed conflicting results for the association between kinesiophobia and the constructs of motor control (Table 6).

### Strength and limitations

The main strength of this study was that we included a comprehensive set of tests to evaluate different constructs of cervical motor control. This study had a larger power to detect important and relevant alterations in cervical motor control and motion compared to other studies in this field. The test setup allowed us to differentiate cervical movement from movement in the thoracic spine, which is important to avoid bias from movement of the torso during tests of cervical ROM and head steadiness. The examiners were not blinded and different examiners performed the data collection in the NP and HC groups, which could have introduced bias. However, standardized instructions were used in all tests to minimize the performance bias. NP subjects performed two and HC subjects one practice trial of the Fly test. A possible learning effect may thus have favored the NP subjects. We did however not find any significant difference between the practice trial and the session used in the data analysis, and thus, we suggest that a possible difference in learning effect between the groups are none or minimal. The HC and the NP subjects performed the same number of trials of the FOE in standing. Since neck pain episodes are frequently reported in non- clinical populations we collected the neck pain history for the HC group. 17 of 91 subjects in the HC group reported one or more previous neck pain episodes, with a median time since last episode of 40 (range 5–120) months. We did a sensitivity analysis were we excluded the 17 subjects and found that the group results for the different tests did not change. Thus, we think that previous episodes of neck pain in the HC group did not bias the conclusions in this study. Other factors like work- load, education and physical fitness may have influenced neck motor control and neck pain and but were not measured in this study.

## Conclusions

NP patients had less cervical ROM, reduced conjunct motion, lower peak velocity during cervical ROM tests, less head motion in the isometric head steadiness tests, showed more “rigid” trajectory head motion patterns and more postural sway in standing on a balance pad. Overall the results clearly suggest altered motor control patterns in subjects with moderate to severe neck pain with long duration characterized by stiffening and rigidity (Table 5). The relationship between different constructs of motor control and clinical features needs further investigation and preferably in a prospective design.

## Additional file

Additional file 1:
**Calibration and data analysis.**

## Abbreviations

CI: Confidence interval
CM: Conjunct motion
EC: Eyes closed
EO: Eyes open
EOB: Eyes open balance pad
ES: Effect size
FOE: Figure-of-eight
HC: Healthy controls
INF: Isometric neck flexion
JPE: Joint position error
NDI: Neck disability index
NP: Neck pain patients
NRS: Numerical rating scale
PCS: Pain Catastrophizing scale
PD: Point deviation
PSEQ: Pain self-efficacy scale
ROM: Range of motion
TSK: Tampa scale of kinesiophobia
TX: Transmitter
