# Supplementary material for: Evidence for a general stiffening motor control pattern in neck pain: a cross sectional study
Source: BMC Musculoskelet Disord. 2015 Mar 17;16:56. doi: 10.1186/s12891-015-0517-2 (PMC4377005; doi:10.1186/s12891-015-0517-2)
Supplement: Additional file 1: — Calibration and data analysis. [file 12891_2015_517_MOESM1_ESM.docx]

**Additional file 1**

**Calibration**

The procedure for calculating the individual coordinate frame for tests of cervical ROM was as follows:

1. The subject was instructed to unrestrictedly perform a full cycle of movement about each axis (i.e. flexion/extension and rotation in the horizontal plane, respectively). The motion data captured during these movements were denoted the *calibration recording*.
2. The dominant axis of rotation of the calibration data corresponding to the flexion/extension movement was calculated. This was done by transforming the corresponding temporal section of the data to rotation matrix form, debiasing the set by multiplying each rotation matrix by the transpose of the set’s cosine average [[1](#_ENREF_1)], and subsequently extracting the 3-vector corresponding to the most dominant principal component for the debiased dataset. This vector was taken as the subject’s flexion/extension axis.
3. The subject’s dominant axis of neck rotation in the horizontal plane was calculated in a similar way based on the calibration data corresponding to the rotation movement. This axis was then adjusted with respect to the subject’s flexion/extension axis by means of Gramm-Schmitt orthogonalization, yielding the subject’s axis of rotation which was now perpendicular to the flexion/extension axis.
4. The axis of lateral flexion was determined as the cross product of the former two (i.e. calculated independent of the calibration recording and the subjects preferred axis of lateral flexion).

**Data analysis**

- Peak velocity was calculated for each primary movement as the peak 3 D angular velocity and expressed as mean of three trials. Here, 3 D velocity was calculated as the rotation velocity at the movement`s instantaneous helical axis.
- Joint position error was calculated as follows. The absolute angular difference (in degrees) between the start and end position of each head rotation was summed over the three cardinal planes of rotation. The mean of the results from the six trials (three to the left and three to the right) was used to express joint position error.

**References**

1. Stavdahl O, Bondhus A, Pettersen K, Malvig K: **Optimal statistical operators for 3-dimensional rotational data: geometric interpretations and application to prosthesis kinematics.** . *Modeling, Identification and Control* 2005, **26**(4):185-200.
